# Oleuropein aglycone stabilizes the monomeric α-synuclein and favours the growth of non-toxic aggregates

**DOI:** 10.1038/s41598-018-26645-5

**Published:** 2018-05-29

**Authors:** Luana Palazzi, Elena Bruzzone, Giovanni Bisello, Manuela Leri, Massimo Stefani, Monica Bucciantini, Patrizia Polverino de Laureto

**Affiliations:** 1 Department of Pharmaceutical Sciences, CRIBI Biotechnology Centre, University of Padova, Padova, Italy; 2 Department of Biomedical, Experimental and Clinical Sciences, University of Firenze, Firenze, Italy; 3 Department of Neuroscience, Psychology, Drug Research and Child Health, University of Firenze, Firenze, Italy

## Abstract

α-synuclein plays a key role in the pathogenesis of Parkinson’s disease (PD); its deposits are found as amyloid fibrils in Lewy bodies and Lewy neurites, the histopathological hallmarks of PD. Amyloid fibrillation is a progressive polymerization path starting from peptide/protein misfolding and proceeding through the transient growth of oligomeric intermediates widely considered as the most toxic species. Consequently, a promising approach of intervention against PD might be preventing α-synuclein build-up, misfolding and aggregation. A possible strategy involves the use of small molecules able to slow down the aggregation process or to alter oligomer conformation favouring the growth of non-pathogenic species. Here, we show that oleuropein aglycone (OleA), the main olive oil polyphenol, exhibits anti-amyloidogenic power *in vitro* by interacting with, and stabilizing, α-synuclein monomers thus hampering the growth of on-pathway oligomers and favouring the growth of stable and harmless aggregates with no tendency to evolve into other cytotoxic amyloids. We investigated the molecular basis of such interference by both biophysical techniques and limited proteolysis; aggregate morphology was monitored by electron microscopy. We also found that OleA reduces the cytotoxicity of α-synuclein aggregates by hindering their binding to cell membrane components and preventing the resulting oxidative damage to cells.

## Introduction

Parkinson’s disease (PD) is one of the most widely occurring neurodegenerative disorders whose symptoms are attributed to the progressive loss of dopaminergic neurons in the *substantia nigra*^1^. The cause of PD is still unknown; however, considerable evidence suggests a multifactorial aetiology involving genetic susceptibility and environmental factors. The main histopathological hallmark of PD is the presence, in the affected neurons, of intracellular inclusions known as Lewy bodies (LB) and Lewy neurites (LN), containing fibrillar aggregates of α-synuclein (Syn)^2^. Syn is implicated at different levels in the pathogenesis of PD and other neurodegenerative conditions, and its aging-related aggregation into amyloid fibrils is a key step in disease aetiology^3–5^. Autosomal dominant forms of PD, resulting from mutations in *SNCA*, the gene encoding Syn, as well as from its duplications and triplications are also known, whereas mutations in a number of different genes are considered risk factors for sporadic PD^6–10^.

**Figure 1:**
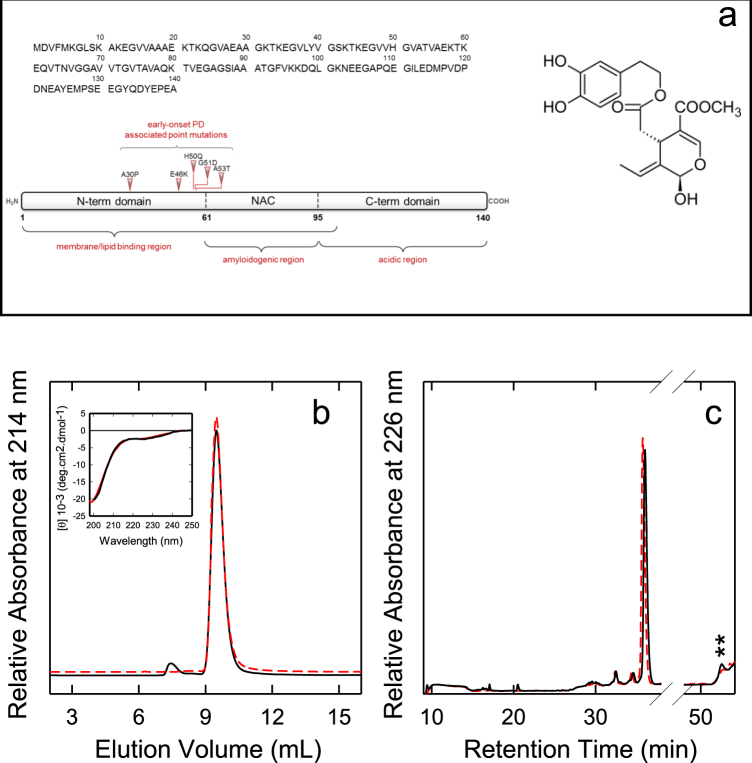
(**a**, left) Amino acid sequence of Syn and scheme of its three major domains: the N-terminal region involved in lipid and membrane binding, containing the mutations associated with familial forms of PD; the NAC domain (61–95) responsible for Syn aggregation properties; the acidic C-terminal region. (**a**, right) Chemical structure of OleA. Interaction between monomeric Syn and OleA. (**b**, inset) Far-UV CD spectra of Syn in the absence (red line) and in the presence of OleA (Syn/OleA 1:10) (black line). Spectra were taken at 25 °C in PBS (pH 7.4). (**b**) Gel filtration and (**c**) RP-HPLC chromatograms of Syn in the absence (red dashed line) and in the presence of 1:10 OleA (black line). In (**c**) the peak relative to RT 51.2 is highlighted with two stars.

Syn is a predominantly presynaptic 140 residues natively unfolded protein^11,12^ whose structure comprises three domains: an N-terminal domain (residues 1–60), a non-amyloid-β component of plaques (NAC) domain (residues 61–95), and a C-terminal domain (residues 96–140) (Fig. 1a, left). Approximately, the first 100 residues, containing 7 imperfect 11-residue repeats, constitute a lipid binding domain^13^. The hydrophobic region spanning residues 61–95 is responsible for the aggregation properties of the protein^14^, that appear to be modulated by the acidic C-terminal tail, containing 15 Glu/Asp residues and many prolines^15^.

Syn is intrinsically disordered in solution^11,12^, but the protein can adopt different types of secondary structure in different environments, as in the presence of lipid micelles, vesicles and membranes, where it acquires α-helical secondary structure^16–19^. Syn interaction with membranes and lipids is considered functionally important^20^; in fact, although its physiological function is not yet fully understood, it has been suggested that Syn may modulate the presynaptic vesicular pool size, promote SNARE-complex assembly^21^, and regulate dopaminergic neurotransmission^22^. The different biological functions of Syn could be a consequence of its conformational flexibility leading to suggest that *in vivo* the protein exists in dynamic equilibrium between different conformational states^23^.

In the LB and LN, Syn is present as highly ordered insoluble polymeric fibrillar aggregates enriched in cross-β-sheet structure^24^. However, several studies have shown that soluble Syn oligomers are the toxic species responsible for disruption of cellular homeostasis and neuronal death through effects on various intracellular targets, including synaptic function^25–27^. Moreover, it is hypothesized a prion-like mechanism by which Syn oligomers can diffuse from one cell to the other, thus spreading the disease through the nervous system^5,26,28^. Increasing evidence indicates that, under pathological conditions, various types of oligomers with different morphologies and physicochemical features are present in tissue^26,27^. Accordingly, Syn oligomers have been generated *in vitro* at different conditions, but it is still unknown whether their structural features and size are similar to those displayed by the species grown *in vivo* or isolated from pathological tissue^28^. Defining the structure of the pathological oligomeric species would be of the utmost importance to understand, at the molecular level, their role in neurodegeneration and in Syn toxicity.

Since the loss of dopaminergic neurons is the primary neuropathological sign of PD, dopaminergic drugs (e.g. levodopa, dopamine agonists and monoamine oxidase, MAO-B, inhibitors) are the main therapeutic options to alleviate the motor symptoms of PD^29^. Levodopa is effective mainly in the early stage of the disease, less as disease progresses. Therefore, other therapeutic strategies, directly targeting Syn, have been proposed^22,30^. Promising approaches are based on the idea to prevent the intracellular and extracellular accumulation of Syn, thus disfavouring its aggregation *in vivo* by inhibiting Syn synthesis using SiRNA^31^ or by increasing its autophagic degradation^22^. It has also been proposed to interfere directly with the aggregation process of Syn by reducing fibrils formation or by stabilizing toxic oligomers confining them in an off-pathway non-toxic or inert form^22,30^.

The use of natural or synthetic small molecules seems to be an attracting way to counteract amyloidogenesis. In particular, plant compounds, such as polyphenols and curcuminoids, have been reported to interfere with the amyloid aggregation of several proteins/peptides of neuropathological interest, including Syn, Aβ and tau^32^. For example, baicalein, curcumin and epigallocatechin 3-gallate (EGCG) have been reported to inhibit Syn fibrillation by stabilizing off-pathway oligomers or by increasing protein solubility and by disrupting long-range interactions that allow a faster reconfiguration rate reducing Syn tendency to aggregate^32–39^. Moreover, NMR studies have led to hypothesize that the production of these non-toxic oligomers is driven by EGCG binding to the Syn region responsible for long-range intramolecular interactions^36,37^.

Oleuropein, the main polyphenol in olive leaves and drupes^40^ is a glycosylated seco-iridoid, which appears mainly in the de-glycosylated (aglycone) form in olive oil (Fig. 1a, right). Oleuropein aglycone (OleA) displays several pharmacological activities including antioxidant, anti-inflammatory, anti-atherogenic, anti-diabetic, anti-cancer, antimicrobial, and antiviral properties^41^ and is commercially available as food supplement or nutraceutical^41^. Recently, it has been reported that OleA interferes with the aggregation *in vitro* of several peptides/proteins associated with amyloid diseases including amylin^42^, Aβ peptides^43^, tau^44^, transthyretin^45^ and beta2-microglobulin (M. Leri *et al*., data not published). It has been proposed that OleA can be beneficial against several amyloid diseases, either systemic (type-2 diabetes)^46^ or neurodegenerative^47^. However, no data are presently available on the interference of OleA with Syn aggregation and its possible protection against the toxicity of Syn aggregates.

In this study, we report that OleA interferes with the path of Syn aggregation and modifies the biophysical properties of preformed Syn assemblies. We also investigated the influence of OleA on the cytotoxicity of variously Syn aggregates and analysed the structural and biological features of the so-generated oligomeric species. Finally, we isolated and characterized an oligomeric species of Syn grown in the presence of OleA.

## Experimental Procedures

### Materials

Oleuropein glycoside was purchased from Extrasynthase and deglycosilated by treatment with almond β-glycosidase (EC 3.2.1.21, Fluka, Sigma-Aldrich) as previously described^42^. Briefly, a 10 mM solution of oleuropein glycoside in 310 μl of 0.1 M sodium phosphate buffer, pH 7.0, was incubated with 8.9 I.U. of β-glucosidase overnight at room temperature. The aglycone (OleA) was precipitated by centrifuging the reaction mixture at 13200 × g for 10 min. The precipitate was suspended in dimethylsulfoxide (DMSO) at 50 mM concentration and batched in aliquots kept frozen and protected from light. Oleuropein deglycosylation was confirmed by mass spectrometry analysis and by assaying the glucose released in the supernatant with the Glucose (HK) Assay kit (Sigma-Aldrich, Saint Louis, MO, USA). Each batch of OleA was used within 1 week once opened. All chemicals were of analytical reagent grade and were obtained from Sigma or Fluka (St. Louis, MO).

### Expression and purification of recombinant human α-synuclein

Syn was expressed in *Escherichia coli* BL21(DE3) cell line transfected with the pET28b/α-syn plasmid. The recombinant protein was expressed and purified according to a previously described procedure^19^ and further purified by RP-HPLC. The identity and purity of the eluted material were assessed by mass spectrometry.

### Aggregation and disaggregation experiments

To induce Syn aggregation, 70 µM protein samples were filtered with a 0.22 µm pore-size filter (Millipore, Bedford, MA, USA) and incubated at 37 °C in PBS (8.0 mM Na_2_HPO_4_, 137 mM NaCl, 2.0 mM KH_2_PO_4_, 2.7 mM KCl, pH 7.4) for up to 7 days under shaking at 500 rpm with a thermo-mixer (Compact, Eppendorf, Hamburg, DE) in the absence or in the presence of OleA (210–700 µM, corresponding to a Syn:OleA molar ratio of 1:3 or 1:10, respectively). Protein aggregation in sample aliquots collected at the indicated incubation times was checked by Thioflavin T (ThT) binding assay, 1-anilinonaphtalene-8-sulfonate (ANS) fluorescence, transmission electron microscopy (TEM), limited proteolysis, gel filtration (GF) chromatography and reverse phase (RP)-HPLC. The interference of OleA in ThT and ANS analysis was assayed by performing controls experiments where the polyphenol was added in pre-made Syn aggregates (48 h and 168 h) either immediately before or after the addition of ThT or ANS solution. Oligomer-enriched or fibril-enriched sample were prepared by incubating Syn for 48 h or up to 168 h, respectively. For disaggregation experiments, 48 h-aged oligomers were incubated with OleA (1:10) at 37 °C under shaking at 500 rpm, for 24 h (Syn 48 h + OleA 24 h) or 5d (Syn 48 h + OleA 5d); 168 h-aged fibrils were incubated with OleA for 8 h (Syn 168 h + OleA 8 h) or for 5d (Syn 168 h + OleA 5d). All aggregate concentrations were expressed as monomer protein concentration.

### Structural characterization

Protein concentrations were determined by absorption measurements at 280 nm using a double-beam Perkin Elmer (Norwalk, CT) Lambda-20 spectrophotometer. The molar absorptivity at 280 nm for Syn was 5960 cm^−1^ M^−1^, as evaluated from its amino acid composition by the method of Gill and von Hippel^48^. Circular dichroism (CD) spectra were recorded by a Jasco (Tokyo, Japan) J-710 spectropolarimeter, using a 1.0-mm path-length quartz cell and a protein concentration of 7.0 µM. The mean residue ellipticity [θ] (degcm^2^ dmol^−1^) was calculated according to the formula [θ] = (θ_obs_/10)(MRW/*lc*), where θ_obs_ is the observed ellipticity in deg, MRW is the mean residue molecular weight of the protein, *l* is the optical path-length (as cm) and *c* is protein concentration (as mg/ml). The spectra were recorded in PBS, pH 7.4. Fluorescence measurements were performed using a Jasco (Tokyo, Japan) FP-6500 spectrofluorimeter and a 0.1-cm path length cuvette. The ThT binding assay was performed accordingly to LeVine^49^ using a 25 µM ThT solution in 25 mM sodium phosphate buffer, pH 6.0. Aliquots (30 µl) of protein samples were collected at the indicated times and diluted into the ThT buffer. Fluorescence emission measurements were performed at 25 °C at an excitation wavelength of 440 nm by recording the ThT fluorescence emission in the 455–600 nm interval. For ANS binding experiments, a 350 nm excitation wavelength was used and the emission spectra were scanned in the 380–600 nm interval^50^. All spectra were recorded at 25 °C using a 50 µM ANS solution and a 2.5 µM protein solution. ANS-binding experiments were carried out in 50 mM Tris-HCl buffer, pH 7.4. ANS concentration was determined using a molar absorption coefficient of 4950 M^−1^ cm^−1^ at 350 nm. To evaluate the possible interference of OleA with the ThT and ANS fluorescence assay, two different controls were performed by monitoring the emission fluorescence spectra intensity of Syn aggregates before and after OleA addition: i) OleA was added together to the probe or ii) OleA was added after the probe and the fluorescence spectra were re-acquired immediately.

### TEM and DLS analysis

Size and morphology of Syn aggregates were investigated by transmission electron microscopy (TEM) and dynamic light scattering (DLS). Negative staining was obtained by placing a drop of the sample solution on a Butvar-coated copper grid (400-square mesh) (TAAB-Laboratories Equipment Ltd, Berks, UK). Then, the sample was dried and negatively stained with a drop of uranyl acetate solution (1.0%, w/v). TEM pictures were taken on a Tecnai G2 12 Twin instrument (FEI Company, Hillsboro, OR) at an excitation voltage of 100 kV. Size distribution of protein samples was calculated on the basis of 382 particles manually extracted from the micrographs using ImageJ software. Only clearly defined spherical and isolated particles were selected. Measurements were obtained with a standard deviation of 5.04. Size distributions were also evaluated by DLS experiments carried out in duplicate, at 25 °C in PBS, pH 7.4, using a Zetasizer Nano-ZS instrument (Malvern Instrument, UK). 12 runs were collected during each measurement.

### Chemical characterization

Gel filtration (GF) chromatography was performed by a Superdex 75 10/300GL column (Amersham Biosciences, Uppsala, Sweden), using an ÄKTA FPLC system (Amersham Biosciences, Uppsala, Sweden). Sample aliquots (200 µL) were taken from the aggregation mixture, filtered (0.22 µm filters), loaded onto the column and eluted at 0.5 ml/min in 20 mM Tris-HCl, 0.15 M NaCl, pH 7.4. The effluent was monitored by recording the absorbance at 214 nm. Column calibration was performed using the following standards: blue dextran (void volume); albumin, 67 kDa; ovalbumin, 45 kDa; α-lactalbumin, 14.4 kDa and aprotinin, 6.5 kDa. The RP-HPLC analyses were carried out on an Agilent 1200 chromatographer (Santa Clara, CA) using a Jupiter C18 column (4.6 × 250 mm; Phenomenex, CA, USA), eluted with the following acetonitrile/0.085% TFA- water/0.1% TFA gradient: 5–25%, 5 min, 25–28%, 13 min, 28–39%, 3 min, 39–43%, 21 min. The effluent was monitored by recording the absorbance at 226 nm. The identity of the eluted material was assessed by mass spectrometry, carried out with an electrospray ionization (ESI) mass spectrometer equipped with a Q-Tof analyzer (Micro) (Waters, Manchester, UK) or a QTof Xevo G2S (Waters, Manchster, UK). Measurements were carried out at 1.5–1.8 kV capillary voltage and 30–40 V cone voltage.

### Limited proteolysis

Limited proteolysis experiments of Syn and Syn/OleA (1:3, 1:10) samples were carried out in PBS, pH 7.4, at room temperature using proteinase K at E/S ratio of 1:1000 (by weight) at 70 µM Syn concentration. The reactions were quenched after 5 min by acidification with TFA in water (4.0%, v/v) and analysed by RP-HPLC, as described above.

### Fingerprinting analysis of off-pathway oligomers

The Syn aggregated species were purified by harvesting the material eluted from RP-HPLC at RT 51.2 min at the conditions described above. The protein samples were dried in Savant and solubilised in 6.0 M guanidine(Gnd)-HCl/20 mM Tris-HCl, pH 8.5. Then, the samples were diluted 1:10 and trypsin was added to an 1:25 enzyme to substrate (E/S) ratio. After 15 h of incubation at 37 °C, the proteolysis mixture was analysed with RP-HPLC by the same column and gradient used for purification and the peptide species were identified by ESI-MS. For proteinase K proteolysis, the sample was further diluted reaching a final concentration of 1.0 M Gnd-HCl before adding the enzyme (E/S 1:1000). The reactions were carried out for 5- or 70-min at 37 °C.

### Cell culture and cell viability assay

SH-SY5Y cells were cultured at 37 °C in complete medium (50% HAM, 50% DMEM, 10% foetal bovine serum, 3.0 mM glutamine, 100 units/ml penicillin and 100 μg/ml streptomycin), in a humidified, 5.0% CO_2_ incubator. All materials used for cell culture were from Sigma. The samples containing aggregating Syn were administered to the cells at a 5.0 μM final concentration. The cytotoxicity of the variously aggregated Syn samples was assessed by the 3-(4,5-dimethylthiazol-2-yl)-2,5-diphenyltetrazolium bromide (MTT, Sigma-Aldrich) reduction inhibition assay based on the protocol described for the first time by Mosmann^51^. In all MTT-experiments, the cells were plated and incubated for 24 h at a density of 10000 cells/well on 96-well plates in 100 μl culture medium. Then, the cells were treated for 48 h with 5.0 µM Syn aggregates grown in the absence or in the presence of OleA (1:10). After exposure to the aggregates, the cells were incubated for 2 h with 100 μl DMEM without phenol red, containing 0.5 mg/ml MTT. Then, 100 μl of cell lysis buffer (20% SDS, 50% N,N-dimethylformamide, pH 4,7) was added to each well and the samples were incubated at 37 °C to allow complete cell lysis. The blue formazan absorbance was recorded at 595 nm with an automatic plate reader (Bio-Rad, Hercules, Cal). Final absorption values were calculated by averaging three independent measurements of each sample after subtraction of the average of the blank solution (100 μl of MTT solution and 100 μl of lysis buffer). All data were expressed as mean ± standard deviation.

### Reactive oxygen species

The intracellular reactive oxygen species (ROS) were determined by monitoring the increase in fluorescence at 538 nm of the fluorescent probe 20,70–dichlorofluorescein diacetate, acetyl ester (CM-H_2_ DCFDA; Sigma-Aldrich), a cell-permeant ROS indicator that becomes fluorescent on removal of the acetate groups by cellular esterases and subsequent oxidation. The SH-SY5Y cells were plated at a density of 10000 cells/well on 96-well plates. The cells were exposed for 48 h to 5.0 μM aggregates, then 10 mM DCFDA in DMEM without phenol red was added. After 30 min, the fluorescence values were detected by Fluoroscan Ascent FL (Thermo-Fisher, Illkinch, France).

### Confocal immunofluorescence

Subconfluent SH-SY5Y cells grown on glass coverslips were treated for 48 h with Syn aggregates at a 5.0 μM final concentration and then washed with PBS. Labelling at the cell surface monosialotetrahexosylganglioside (GM1) was obtained by incubating the cells with 10 ng/ml CTX-B Alexa488 in complete medium for 30 min at room temperature. Then, the cells were fixed in 2.0% buffered paraformaldehyde for 10 min, permeabilized by a cold 1:1 acetone/ethanol solution for 4 min at room temperature and blocked with PBS containing 0.5% BSA and 0.2% gelatine for 30 min. After blockage, the cells were incubated for 1 h at room temperature with a rabbit polyclonal anti-Syn antibody (Abcam, Cambridge UK) diluted 1:500 in blocking solution and then washed with PBS for 30 min under stirring. The immunoreaction was revealed by Alexa 568-conjugated anti-rabbit Abs (Molecular Probes, Eugene, Oregon, USA) diluted 1:100 in PBS. Finally, the cells were washed twice in PBS and once in water to remove non-specifically bound Abs. Cell fluorescence was imaged using a confocal TCS SP5 scanning microscope (Leica) equipped with a HeNe/Ar laser source for fluorescence measurements. Sample observations were performed by a Leica Plan Apo X63 oil immersion objective suited with optics for differential interference contrast acquisition. FRET analysis was carried out by the FRET sensitized emission method as previously reported^52^.

### Data analysis and statistics

Statistical analysis was performed using Student’s t-test. The results were compared using Student’s *t*-test between two groups, *P < 0.05; **P < 0.01; ***P < 0.001 versus untreated cells and °P < 0.05; °°P < 0.01; °°°P < 0.001 versus cells treated with aggregates grown in the absence of OleA.

## Results

### Interaction between OleA and monomeric Syn

The effect of OleA on the secondary structure of Syn was evaluated by far-UV CD spectroscopy (Fig. 1b, inset). We found that at neutral pH the protein displays a far-UV CD spectrum typical of a substantially unfolded polypeptide chain, with an intense minimum near 198 nm and the absence of characteristic bands in the 208–230 nm region (Fig. 1b, red dashed line). In the presence of OleA at two different Syn/OleA molar ratios (1:3 or 1:10), no changes in the shape or intensity of the spectra recorded just after sample preparation were detected, supporting the lack of any direct conformational modification. For simplicity, Fig. 1b, inset, reports only the spectrum relative to the 1:10 Syn/OleA ratio (black continuous line). To confirm this, we also analysed Syn in the absence and in the presence of OleA (1:10) by gel filtration (GF) (Fig. 1b) and RP-HPLC (Fig. 1c). To avoid technical problems, the samples for GF analysis were filtered (0.22 µm filters) before loading; to simplify the figure, the profiles obtained in the presence of 1:3 Syn/OleA ratio are not shown. The GF profiles of Syn in the absence or in the presence of OleA are similar and show a major species eluting at ~9.4 ml, corresponding to monomeric Syn. In the presence of OleA a minor high MW species (~1%) at shorter elution volume was also seen (Fig. 1b). No variation in the retention time (RT 35.5 min) of Syn when eluted in the presence of OleA was seen in RP-HPLC; however, we noticed the presence of a new peak at RT 51.2 min (Fig. 1c, highlighted with two stars), indicating increased hydrophobicity, likely due to some chemical modifications or change in the aggregation state of this species. These two main fractions were collected and analyzed in more detail. Mass spectrometry analysis indicated the presence of Syn species in both fractions (Table 1). Syn appeared chemically modified in very low percentage (~5.0%) by the presence of some covalent adducts resulting in mass increases of 205 and 223 Da. These adducts could result from the reaction of Syn with demethylated and dehydrated forms of the elenolic acid arising from OleA degradation^53^. The presence of these modifications in both monomeric and late-eluting Syn allows to exclude they are the cause of the RP-HPLC shift.

**Table 1:** Chemical characterization of the main protein species in the mixture formed by Syn and OleA in the 1:10 ratio eluted from the RP-HPLC column (Fig. 1c).

RT^a^ (min)	Found mass^b^ (Da)	Theoretical mass^c^ (Da)	Protein species
35.5	14461.5 (± 0.1) 14477.2 (± 0.8) 14682.8 (± 0.8)	14460.5 14476.5 14683.2	Syn Syn + 1ox Syn + 223
51.2	14462.1 (± 1.5) 14478.5 (± 1.8) 14665.2 (± 0.5) 14683.2 (± 0.1)	14460.5 14476.5 14665.2 14683.2	Syn Syn + 1ox Syn + 205 Syn + 223

The term ox indicates an oxidized derivative (+16 Da).

^a^Proteins are listed in order of retention time (RT).

^b^Experimental molecular masses determined by ESI-QTOF-MS.

^c^Molecular masses calculated from Syn amino acid sequence.

### OleA interferes with Syn aggregation into amyloid fibrils

**Figure 2:**
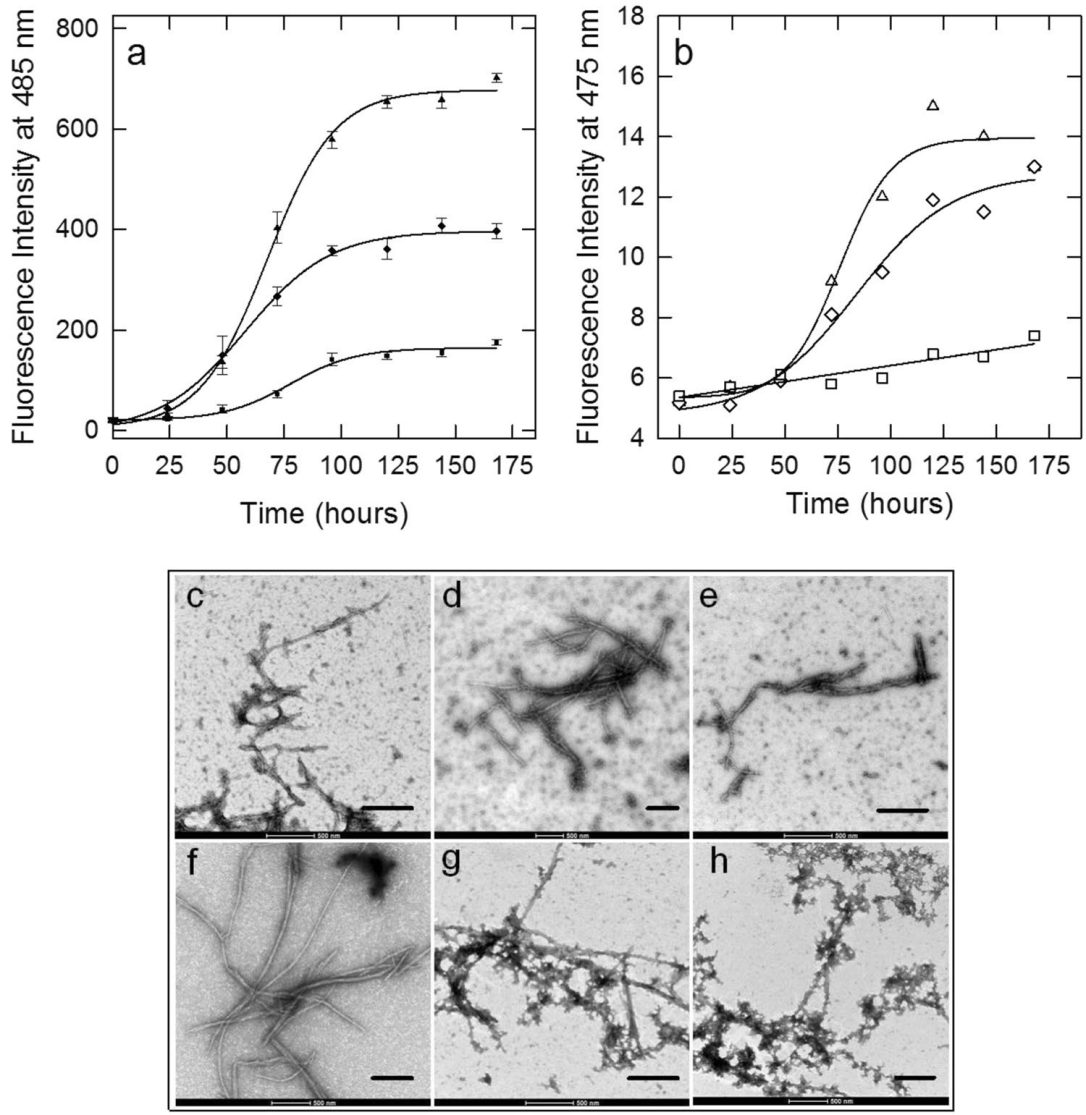
Effect of OleA on Syn aggregation probed by ThT (**a**), ANS (**b**) fluorescence spectroscopy and TEM (**c**–**h**). Syn aggregation kinetics (triangle), Syn aggregation in the presence of 1:3 (diamond) or 1:10 (square) OleA. For the ThT assay, aliquots (30 μl) of the protein solution, incubated for the indicated times, were added to a 25 µM solution (470 μl) of ThT in 25 mM phosphate buffer, pH 6.0. Excitation wavelength: 440 nm, fluorescence emission: 485 nm. For the ANS assay, excitation wavelength: 350 nm, fluorescence emission: 475 nm at 25 °C. TEM pictures taken from Syn aggregation mixture after 48 h and 168 h of incubation in the absence (**c**,**f**) or in the presence of a 1:3 (**d**,**g**) or 1:10 (**e**,**h**) Syn/OleA ratio. Scale bar in every picture corresponds to 500 nm.

When incubated *in vitro* at 37 °C and pH 7.4 for 168 h, Syn aggregates into fibrils^12^. The kinetics of amyloid fibril formation were monitored by following the increase of ThT fluorescence emission at 485 nm during the aggregation process. According to previous data^54,55^, we found an initial lag phase followed by an exponential growth phase and a final plateau, consistent with a nucleation-dependent mechanism (Fig. 2a). TEM imaging showed that the 48 h sample was populated with oligomers and pre-fibrillar aggregates (Fig. 2c), while fibrils with typical amyloid morphology became the major aggregated species after 168 h of incubation (Fig. 2f). Then, we investigated the effect of OleA on the kinetics of fibril formation by using a Syn/OleA ratio of 1:3. We found that the curve maintained a sigmoid trend, yet in the presence of OleA (Fig. 2a), and some amyloid fibrils were still detectable in the sample (Fig. 2d,g). When we used a 1:10 Syn/OleA ratio, the ThT fluorescence intensity was scarcely increased (Fig. 2a) but other types of aggregates populated the sample, as seen by TEM imaging (Fig. 2e,h), suggesting some interference of the polyphenol with the aggregation process.

Syn aggregation was further characterized in terms of hydrophobic exposure, monitored as ANS binding, by recording the increase of fluorescence emission at 475 nm by ANS added to sample aliquots withdrawn from the aggregation mixture at different incubation times (Fig. 2b). The experimental data points fit an irregular sigmoidal curve recalling, in part, the trend observed in the ThT experiment. When the aggregation was carried out at Syn/OleA 1:10, we found a moderate increase of fluorescence intensity during the process. Overall, these data confirm that OleA interferes with the aggregation process of Syn.

### Mapping α-synuclein conformation during aggregation in the presence of OleA by limited proteolysis

**Figure 3:**
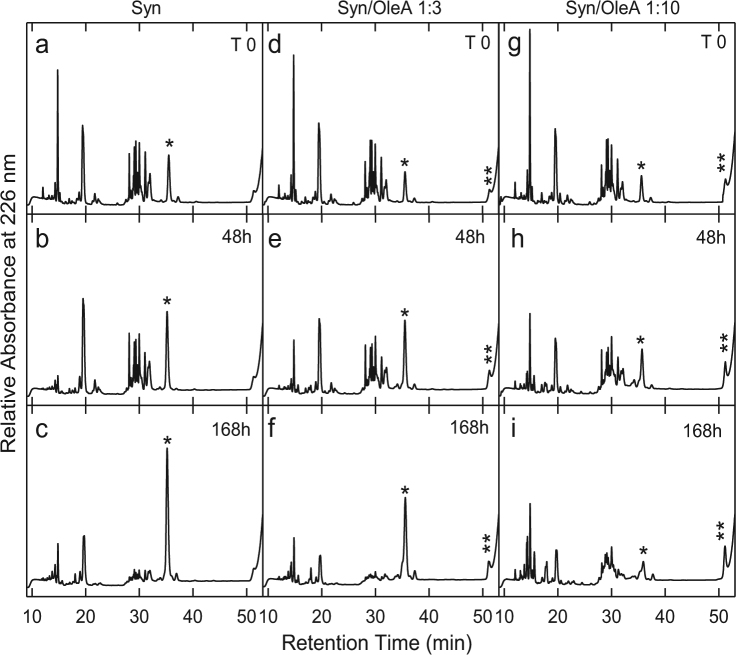
Syn aggregation in the absence or in the presence of OleA 1:3 and 1:10 ratio investigated by limited proteolysis. During aggregation, proteinase K was added at an E/S ratio of 1:1000 (by weight) to aliquots (40 µl) taken from the mixture at different time intervals (0, 48, 168 h). Proteolysis was allowed to take place at 22 °C for 5 min and then quenched by addition of 4.0% TFA in water. The proteolysis samples were analysed by RP-HPLC using a Jupiter C18 column (4.6 × 250 mm) and eluted with a gradient of acetonitrile/0.085% TFA vs water/0.1% TFA from 5.0% to 25% in 5 min, from 25% to 28% in 13 min, from 28% to 39% in 3 min, from 39% to 43% in 21 min. The peaks relative to intact Syn and to the oligomeric species are highlighted.

The propensity of Syn to be hydrolysed by proteinase K at different stages of amyloid aggregation was investigated by highlighting the sites of proteolysis in the absence or in the presence of OleA. Proteinase K was chosen because of its broad specificity^56^; accordingly, its cleavage sites are not expected to be dictated by the amino acid sequence, rather, they depend on the conformational and dynamical features of the polypeptide chain^57,58^. An E/S ratio of 1:1000 was used in the experiment and the proteolysis mixtures were analysed by RP-HPLC after 5 min incubation of Syn with the protease (Fig. 3). The sites of proteolysis were determined by mass spectrometry analysis of the isolated HPLC fractions (Table S1). As expected from its natively unfolded character, monomeric Syn was highly susceptible to the proteolytic attack and many fragments were seen in the chromatogram after 5 min-incubation (Fig. 3a). When Syn was kept for 48 h in aggregation buffer, a slightly reduced susceptibility to proteolysis was evident (Fig. 3b); moreover, at longer incubation times, Syn became progressively more resistant to proteolysis, in parallel with the increase of fibril content in the aggregation mixture, as previously observed for other proteins^58^ (Fig. 3c). Then, the proteolysis experiment was carried out on the Syn/OleA 1:3 or 1:10 samples. We found that at time 0 all proteolytic mixtures showed a similar pattern (Fig. 3a,d,g). As the aggregation time increased, Syn appeared apparently less resistant to proteolysis (Fig. 3e,f,h,i); in fact, the peak relative to the intact protein (RT 35.5 min) was slightly less intense than that obtained in the absence of OleA (highlighted with a star in the figure). It is worth noting the presence of the peak at RT 51.2 min (highlighted with two stars in Fig. 3). This species increased as far as the aggregation time was prolonged (from T 0 to T 168 h,) and OleA concentration in the sample was increased (Fig. 3d–f,g–i).

Overall, the proteolysis data indicate that the presence of OleA in the aggregation mixture of Syn increases the monomeric Syn sensitivity to proteolysis. Moreover, OleA favours the growth of other types of aggregated species, that elute late in RP-HPLC and exhibit increased resistance to proteolysis. To better evaluate the relative amount of each population, the area of the peaks relative to the material obtained by PK digestion at different times of aggregation was calculated (Fig. S3) and commented in the Discussion.

### Characterization of the off-pathway species arising during Syn aggregation in the presence of OleA

**Figure 4:**
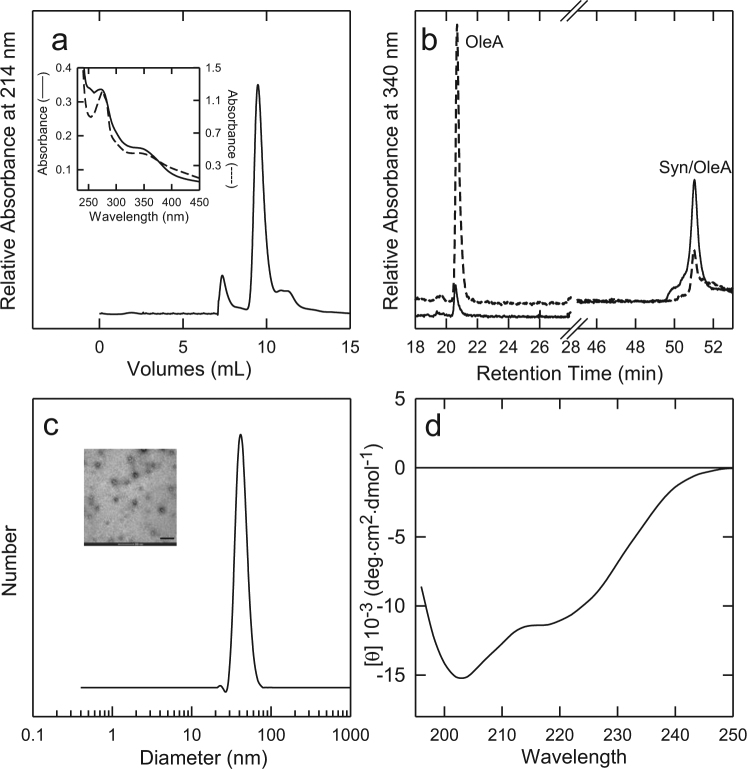
Characterization of Syn off-pathway oligomers (RT 51.2) grown in the presence of OleA. (**a**) Gel filtration chromatography of the Syn/OleA (1:10) mixture after 168 h of incubation Inset: UV-vis spectra of the fractions at 7.4 (−) and 9.4 (−) mL of elution volume in GF. (**b**) RP-HPLC chromatogram at 340 nm of the Syn/OleA (ratio 1:10) mixture at time 0 (dashed line) and 168 h (continuous line) of incubation. (**c**) DLS and TEM (inset) analysis of the purified oligomers. (**d**) Far-UV CD of the purified oligomers. The oligomers were purified by RP-HPLC and concentrated until organic solvent and TFA removal, but never dried to avoid morphological changes. Other experimental details are reported in the methods section. The scale bar in the TEM image correspond to 200 nm.

Next, we look at the fraction eluted at RT 51.2 min by RP-HPLC under aggregation conditions in the presence of OleA. This species was collected and concentrated for further characterization. Figure 4a reports the GF chromatogram relative to the Syn/OleA (1:10) mixture after 168 h of aggregation. Two main fractions (7.4 ml; 9.4 ml) were detected. The species eluting at 7.4 ml and increasing over time (cfr. Fig. 1b) was related to that eluting at 51.2 min in RP-HPLC, when an aliquot of the corresponding GF fraction was reloaded onto the RP-HPLC column (not shown). Its UV-vis spectrum (Fig. 4a, inset, continuous line) showed a profile characterized by two main signals at 340 nm and 280 nm, indicative of the presence of both OleA and Syn. The UV-Vis spectrum of OleA alone obtained just after sample preparation and after 168 h of incubation was shown in Fig. S1b, as a reference. The fraction at 9.4 ml, eluted as monomeric Syn, also contained OleA, whose absorbance bands were detectable in the UV-vis spectrum (Fig. 4a, inset, dashed line).

The same mixture (Syn/OleA, 1:10 ratio; 168 h) was also analysed by RP-HPLC by recording the effluent at 340 nm (Fig. 4b) to monitor OleA absorbance. The chromatogram relative to the analysis of the freshly prepared Syn/OleA mixture is also reported as a reference (dashed line). The chromatograms were characterized by two main peaks at RT 20.5 min and RT 51.2 min, respectively. These fractions were analysed by MS, confirming the presence of OleA in both and the absence of protein material in the peak at 20.5 min (not shown). The intensity of the peaks at 20.5 min and 51.2 min reciprocally changed during incubation. At the beginning, the signal relative to OleA was higher and decreased with time, probably due to the formation of the OleA-Syn complex eluting at 51.2 min. When imaged by negative stain TEM, the latter sample showed a morphology consistent with an oligomeric structure (Fig. 4c, inset). These oligomers appeared as not fully homogenous spheroidal particles with an average diameter of 22.5 nm (12.5 nm the smaller species, 42.3 nm the larger ones). The largest aggregates were not included in these measurements. By DLS analysis, the average hydrodynamic diameter was of ~43 nm (Fig. 4c).

The far-UV CD spectrum of the RT 51.2 sample is reported in Fig. 4d. The shape of the spectrum and the presence of two minima at ~203 and ~220 nm indicate that oligomer structure is not disordered, when compared to that resulting from the CD spectrum of monomeric Syn (Fig. 1c, inset). The spectra also showed that different conformers, partially folded and unfolded, did exist at equilibrium. Further characterization is required to better define the secondary structure content of these species.

**Figure 5:**
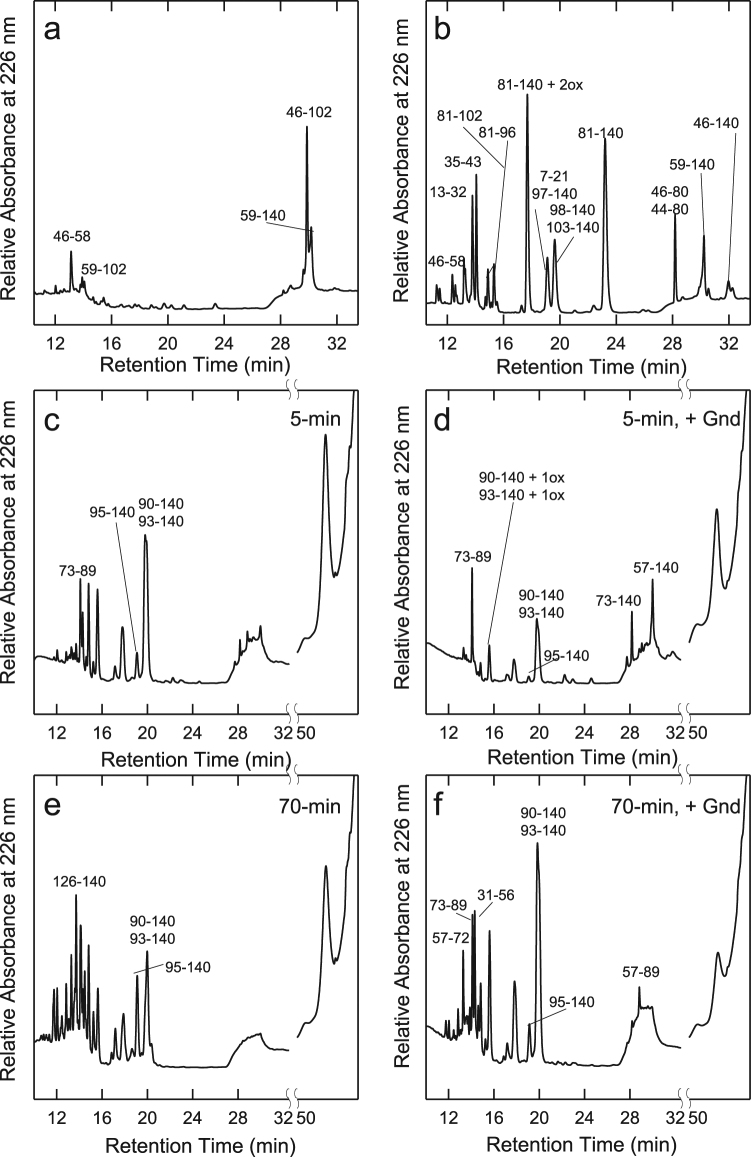
Mapping of (**a**) Syn off-pathway oligomers (RT 51.2) and (**b**) Syn monomers by trypsin proteolysis. The chromatograms refer to incubation with PK of the RT 51.2 sample for 5-min (**c**,**d**) or 70 min (**e**,**f**). In (**d**) and (**f**), the proteolysis was carried out on samples partially destabilized in Gnd-HCl. The numbers close to the peaks indicate the identified fragments. The term ox refers to the presence of oxidation in the peptides.

Then, we mapped the overall structure of the oligomeric species at RT 51.2 min by finger printing analysis using trypsin after partial destabilization of the complex by Gnd-HCl (Fig. 5). Indeed, these oligomers were quite resistant to proteolysis, probably because of some protection by OleA or by OleA-induced conformational modifications (cfr. Fig. 3). By performing exhaustive fragmentation by trypsin (15 h), only a few peptide species were found in the proteolysis mixture of the RT 51.2 species (Fig. 5a), when compared with same analysis carried out with monomeric Syn (Fig. 5b); at variance, fragments encompassing the entire sequence were detected in the latter (Table S2). In particular, the large fragments corresponding to the sequences 59–140 and 46–102 remained undigested even after 15 h (Fig. 5a) of incubation in the proteolysis mixture, suggesting that these species are rigid, structured or protected. To explain the presence of these peptide species, a pattern of fragmentation can be hypothesized and it is conceivable that RT 51.2 was cleaved, initially, at the level of the 45–46 peptide bond, thus generating the 1–45 and 46–140 fragments. The N-terminal species 1–45 was not visible, since it was further fragmented into smaller fragments. The C-terminal 46–140 was further cleaved at the level of the 102–103 peptide bond, producing the quite resistant 46–102 fragment and the 103–140 fragment, that was further degraded in smaller species. On the other hand, the 59–140 fragment was also seen in the chromatogram. This species could arise from the cleavage of the 58–59 peptide bond in the 46–140 fragment. Then, the 59–140 species underwent further fragmentation generating the 59–102 species. In conclusion, the 46–102, and in minor extent the 59–140, are the most resistant, upon prolonged trypsin treatment and after oligomer destabilization by Gnd-HCl.

A better structural characterization of the off-pathway oligomers (RT 51.2) was obtained by carrying out the mapping by PK with (Fig. 5d,f) or without (Fig. 5c,e) destabilization by Gnd-HCl. Indeed, the rationale of this approach was based on the general observation that, in the absence of the denaturant, the substrate of the protease is the most flexible species in equilibrium with a more structured one, not accessible by PK. Gnd-HCl addition destabilized the PK-resistant species, making it more susceptible to proteolysis. Moreover, in this case, the proteolysis reactions were carried out for 5- and 70-min, to identify the first proteolytic events (Fig. 5c,d) and those occurring subsequently on a perturbed substrate, in order to recognise the proteolysis resistant species accumulating in the mixture (Fig. 5e,f). In the absence of Gnd-HCl, the RT 51.2 species was only partially fragmented by the protease, as indicated by the fact that its peak was still visible even after 70 min of reaction (Fig. 5e) and that any peculiar fragment did not accumulate in the mixture. However, following partial destabilization of the complex, this species was almost exhaustively digested upon incubation up to 70 min (Fig. 5f). Moreover, in the presence of Gnd-HCl, after 5 min some species seemed to accumulate and two main cleavages did occur (Fig. 5d). The first, involving the 56–57peptide bond, generated the 1–56 and 57–140 fragments, whereas the other, involving the 72–73 peptide bond, produced the 1–72 and 73–140 fragments (Table S3). The two N-terminal fragments (1–56 and 1–72) were scarcely represented in comparison to the C-terminal ones (57–140 and 73–140) since the former were probably cleaved into smaller hydrophilic fragments. After longer incubation with the enzyme (Fig. 5f, 70 min), further cleavages took place on the C-terminal species, which generated mainly the 57–89, 73–89 and 90–140 fragments, that remained undigested even after 70 min of incubation with PK (Fig. 5f). These data confirm those obtained by trypsin and suggest the coexistence of two different population of off-pathway oligomers: one more susceptible to proteolysis, undergoing fragmentation in the absence of denaturant, and another, more resistant, that is cleaved only after denaturation. Moreover, from a comparison between the two proteolytic patterns, those encompassing residues 46–102, 57–89, 59–140 and 90–140 appear to be the more protected regions, belonging to the NAC and C-terminal regions of the Syn sequence.

### Syn aggregates grown in the presence of OleA show reduced cytotoxicity

**Figure 6:**
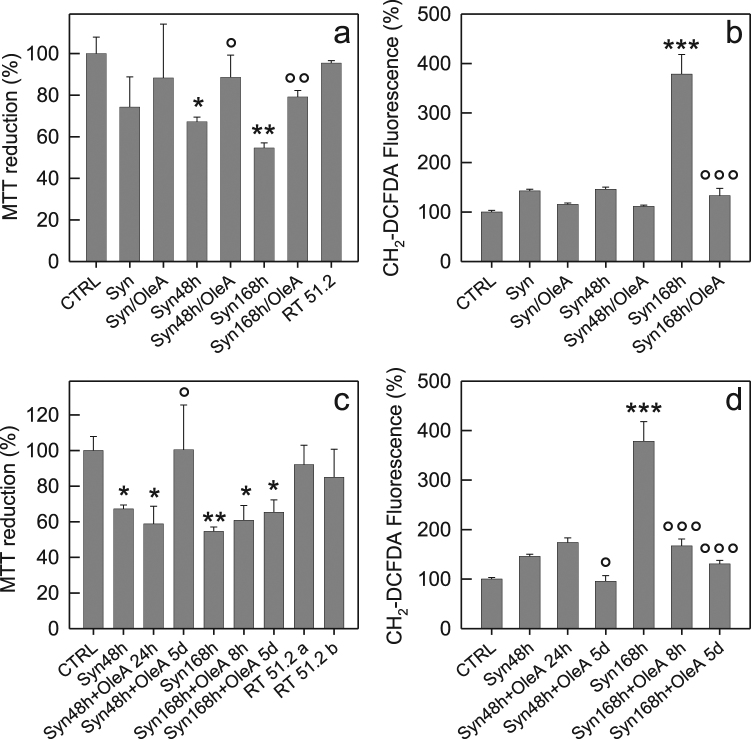
Cell viability and ROS levels. (**a**,**b**) SH-SY5Y cells treated for 48 h with a 5.0 μM Syn solution obtained at different times of aggregation (0, 48 and 168 h) enriched in monomeric (Syn), oligomeric (Syn 48 h) or fibrillar (Syn 168 h) species, respectively. Each sample was obtained in the absence or in the presence of OleA (Syn:OleA, 1:10). (**a**) Cell viability assessed by the MTT reduction assay and (b) by ROS production. For the MTT assay, the RT 51.2 species purified by RP-HPLC was also tested. (**c**,**d**) OleA modulates the toxicity of preformed Syn assemblies. (**c**) MTT assay and (**d**) ROS endogenous levels in SH-SY5Y cells exposed for 48 h to 5.0 μM Syn enriched in oligomers (Syn 48 h) or fibrils (Syn 168 h). The oligomeric samples were also analysed following addition of OleA after further incubation of 24 h or 5 d (Syn 48 h + OleA 24 h) and (Syn 48 h + OleA 5d). The fibrillar species were analysed after 8 h or 5 d of incubation in the presence of OleA (Syn 168 h + OleA 8 h) and (Syn 168 h + OleA 5d). The RT 51.2 samples purified by RP-HPLC from the mixture corresponding to (Syn 48 h + OleA 24 h) sample (RT 51.2a) and from that corresponding to (Syn 168 h + OleA 8 h) sample (RT 51.2b) were also tested by the MTT assay. Error bars indicate the standard deviation of independent experiments carried out in triplicate. Statistical analysis was performed using Student’s t-test. The results were compared using Student’s *t*-test between two groups, *P < 0.05; **P < 0.01; ***P < 0.001 versus untreated cells and °P < 0.05; °°P < 0.01; °°°P < 0.001 versus treated cells with Syn aggregates grown without OleA.

Once described at the molecular level the interference of OleA with Syn aggregation, we investigated whether OleA also affected aggregate cytotoxicity. A first experiment was designed to determine the cytotoxicity of Syn aggregates at different fibrillation times. To this purpose, we used human neuroblastoma, SH-SY5Y, cells exposed for 24 or 48 h to Syn solutions enriched of protein in different conformations: monomer, oligomers or fibrils, obtained by incubating the protein for 0, 48 or 168 h in aggregation conditions, respectively. Oligomers and fibrils were dose-dependently cytotoxic to cells exposed for 48 h, as shown by the MTT assay (Fig. 2S). Under these conditions, the solution enriched in Syn fibrils (Syn 168 h) was the most cytotoxic sample, followed by the solution enriched in oligomers (Syn 48 h); some cytotoxicity, not dose-dependent, was also seen in the case of the monomeric protein before aggregation started (Syn) (Fig. 2S). Based on these data, we decided to expose the cells for 48 h to the three different Syn solutions (5.0 μM) enriched in monomers, oligomers or fibrils, in the absence or in the presence of 1:10 Syn/OleA. The MTT assay showed a significant decrease of toxicity when the cells were treated with samples aggregated for 48 or 168 h in the presence of OleA (Syn 48 h/OleA, Syn 168 h/OleA) (Fig. 6a). Cell survival after exposure to the Syn 48 h/OleA was around 100% ± 10.6 while cell survival was around 80% ± 3.1 in the presence of the Syn 168 h/OleA indicating that OleA was only partially effective in protecting cells against fibril cytotoxicity. Furthermore, the evaluation, by MTT assay, of the toxic power of the species eluted in RP-HPLC at RT 51.2 min showed that this sample was completely harmless even when tested purified at the final concentration of 5.0 μM (Fig. 6a).

In consideration of the key role performed by oxidative stress in the pathogenesis of PD, we also sought to assess the redox status of the variously treated cells by using the ROS-sensitive fluorescent probe CM-H_2_ DCFDA. We found that the different Syn aggregates stimulated some ROS production in SH-SY5Y cells that was particularly evident when the cells were exposed for 48 h to Syn fibrils (Syn 168 h) (Fig. 6b). However, cell exposure to the different aggregates of Syn grown in the presence of 1:10 Syn/OleA did not result in any statistically significant increase of ROS levels (Fig. 6b). These data led us to conclude that the presence of OleA during Syn aggregation decreases the ability of the resulting aggregates to raise ROS production thus making non-toxic the oligomers whereas the toxicity of the fibrillar sample was not completely abolished.

### OleA effect on pre-formed Syn oligomers and fibrils

**Figure 7:**
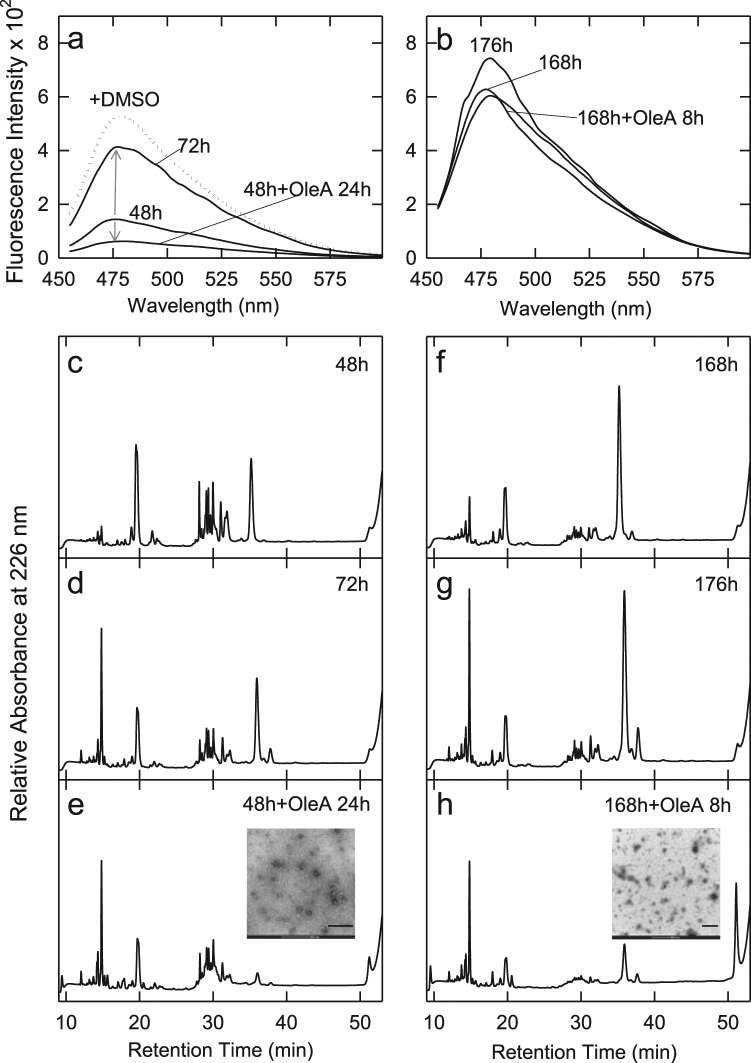
OleA-induced Syn disaggregation probed by (**a**,**b**) ThT fluorescence and (**c**–**h**) limited proteolysis. During aggregation, aliquots were collected from the mixture at 48 h and 168 h, then OleA was added to a 1:10 final Syn:OleA ratio. ThT spectra in (**a**) refer to Syn samples corresponding to 48 h of aggregation (48 h) and to further 24 h of incubation in the absence (72 h) or in the presence (48 h + OleA 24 h) of OleA. The ThT spectra in (**b**) were recorded at 168 h and after additional 8 h of incubation without (176 h) or with (168 h + OleA 8 h) OleA. (c-h) RP-HPLC analysis of the proteolysis mixture of Syn samples during aggregation (**c)** at 48 h, (**d**) after further 24 h of incubation in buffer without polyphenol (72 h), (**e**) in the presence of OleA. The same scheme was followed for Syn samples collected from the aggregation mixture at 168 h of incubation (**f**), after further 8 h of incubation in the absence (**g**) or in the presence (**h**) of OleA. (**e**,**h**, inset), TEM images taken from samples corresponding to the peaks eluting at RT 51.2 min; scale bar: 200 nm.

Once established that OleA interferes with Syn aggregation yielding substantially non-toxic aggregates, we analysed the effect of OleA on preformed Syn oligomers and fibrils to assess whether it favoured fibrils disassembly with leakage of toxic oligomers. OleA (1:10 ratio) was added to preformed solutions enriched in oligomeric species (48 h) or fibrils (168 h). The disaggregation activity by OleA was followed by fluorescence spectroscopy, limited proteolysis and MTT assay. The ThT spectra (Fig. 7a) were obtained from Syn oligomers (48 h) after further 24 h of incubation in the presence (48 h + OleA 24 h) or in the absence (72 h) of OleA in comparison with Syn oligomers (the starting point) (48 h). As expected, in the absence of OleA, the aggregation process proceeded towards fibrils formation with substantial increase of ThT intensity (as indicated by the arrow). The 48 h + OleA 24 h sample showed a decrease of ThT fluorescence, indicative of a stop or a slowdown of the formation of Syn aggregates. The same experimental scheme was followed with Syn fibrils samples (168 h). In this case, ThT fluorescence spectra were recorded after further 8 h of incubation with (168 h + OleA 8 h) or without OleA (176 h). We did not find any remarkable reduction of ThT fluorescence in the (168 h + OleA 8 h) sample, which indicated a negligible effect of OleA on preformed fibrils.

The same samples were also subjected to limited proteolysis (Fig. 7c–h). As expected, the sample aged for 72 h in the absence of OleA (Fig. 7d) showed a slightly increased resistance to proteolysis in comparison to the sample aged for 48 h (Fig. 7c) and small fragments eluting at short RT appeared in the chromatogram. In the proteolytic pattern obtained from samples treated with OleA for 24 h (48 h + OleA 24 h), the low intensity of the peak relative to intact Syn (RT 35.5 min) suggested that the monomeric Syn still present in the mixture was fragmented and resulted, under these conditions, more susceptible to proteolysis (Fig. 7e). In the meantime, the species at RT 51.2 min, previously found to increase as far as the aggregation time was prolonged, appeared in the chromatogram. In synthesis, monomeric Syn underwent formation of a species more susceptible to proteolysis (the peak of intact Syn was decreased) and a protease-resistant one (RT 51.2).

The proteolytic patterns relative to the aggregation mixture at 168 h and 176 h, in the absence of OleA, are quite similar since no critical events occurred in this time (Fig. 7f,g). However, upon addition of OleA (Fig. 7h), the proteolytic pattern was completely different and the RT 51.2 species arose in higher percentage (Fig. S4). Also in this case, the proteolysis data can be explained with the co-existence, in the mixture, of species characterized by different susceptibility to proteolysis. The species eluting at RT 51.2 min, an aggregated and highly hydrophobic one induced by the presence of OleA either during aggregation or at the end of the latter, was accumulating in the mixture and represented a PK- resistant fraction.

We also investigated whether OleA modified the toxicity of Syn preformed oligomers and fibrils. To this aim, we compared the (Syn 48 h + OleA 24 h) and (Syn 168 h + OleA 8 h) cytotoxicity to SH-SY5Y cells with that displayed by the same samples without the polyphenol by using the MTT and ROS assay. In addition, Syn oligomers and fibrils were incubated in the presence of OleA for an extra time (5 days), to assess whether OleA had some effects under extreme experimental conditions. Figure 6c,d show that the (Syn 48 h + OleA 24 h) sample maintained its potential toxicity; however, a prolonged OleA treatment (Syn 48 h + OleA 5d) made it harmless to cells (Fig. 6c) that also displayed reduced endogenous ROS levels (Fig. 6d). As far as the effect of OleA on preformed fibrils is concerned, we observed a very slight decrease of fibril cytotoxicity in both samples (Syn 168 h + OleA 8 h) and (Syn 168 h + OleA 5d) (Fig. 6c). In spite of an evident cell sufferance, the fibrils treated with OleA for short and long incubation times, induced a significant decrease in ROS intracellular levels (Fig. 6d). We also evaluated the toxicity of the RT 51.2 samples after its purification by RP-HPLC from the mixture corresponding to the Syn 48 h + OleA 24 h sample (RT 51.2a) and from that corresponding to the Syn 168 h + OleA 8 h sample (RT 51.2b). The MTT assay confirmed that this sample was completely innocuous to SH-SY5Y cells (Fig. 6c).

### OleA interferes with the interaction of Syn aggregates with the cell membrane

Finally, we investigated whether OleA interfered with the ability of Syn aggregates to interact with the cell membrane. In fact, recent data have widely reported that amyloid aggregates are able to bind to lipid bilayers and that aggregate interaction with the cell membranes is a crucial step of amyloid cytotoxicity^59,60^. In particular, it has been reported that exogenous oligomers and fibrils bind to the cell membrane and accumulate in lipid rafts, liquid-ordered membrane microdomains rich in functional proteins (receptors, carriers, signalling molecules), sphingosine, cholesterol and ganglioside GM1^61,62^. In this context, Syn oligomers have been reported to display membrane-disrupting power on anionic lipid bilayers and to alter the permeability and the integrity of the cell membrane^63^.

**Figure 8:**
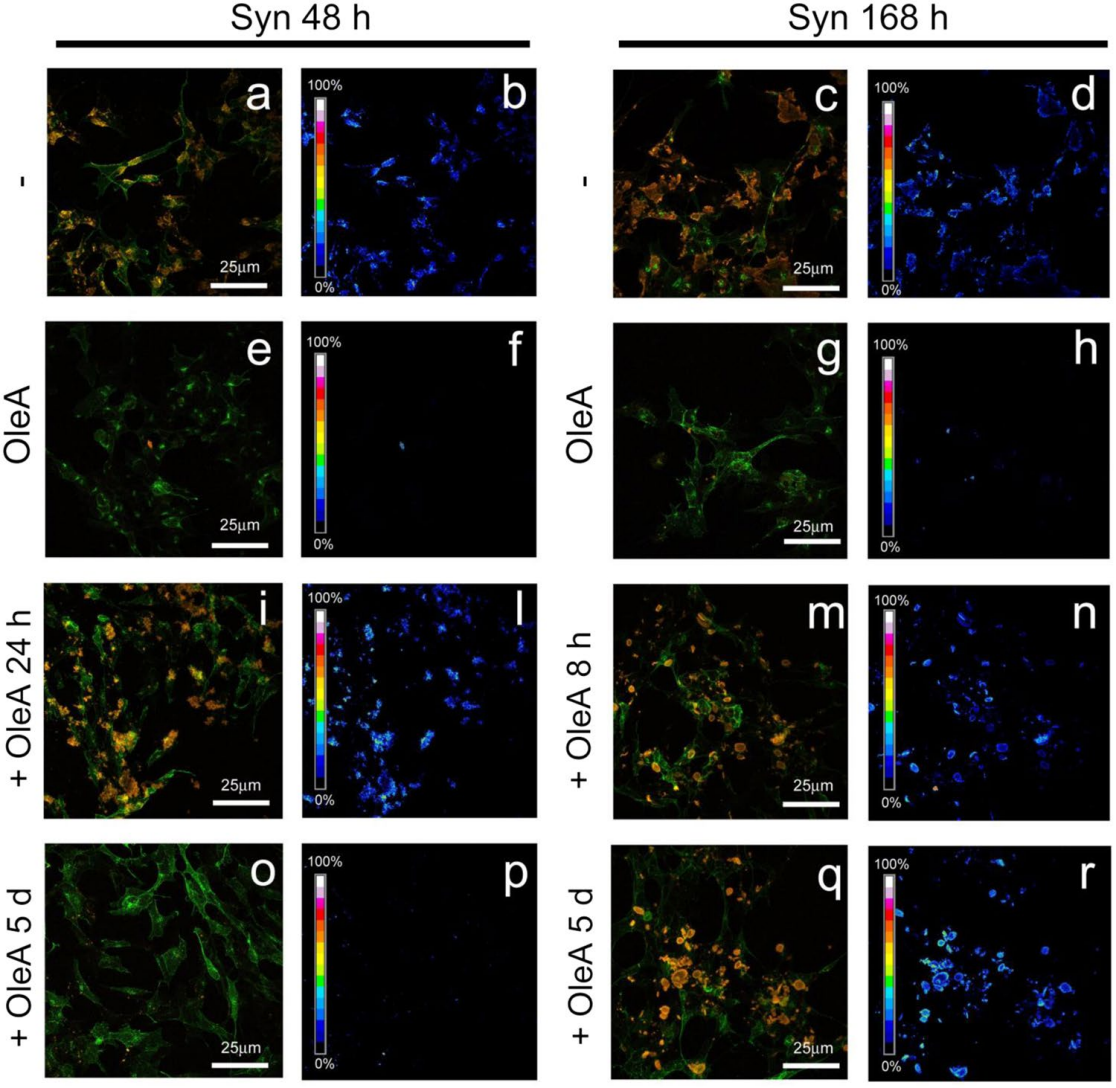
The complex OleA-Syn does not bind to the plasma membrane. (**a**,**e** and **c**,**g**) Z-projection of Syn samples grown in the presence (Syn 48/OleA, Syn 168/OleA) or in the absence of OleA (Syn 48 h, Syn 168 h) by immunostaining (red) and GM1 staining (green) on SHSY5Y cell membrane. (**b**,**f** and **d**,**h**) FRET analysis between GM1 staining and aggregate immunostaining. OleA affects preformed Syn assemblies and their membrane interaction. (**i**,**o**, and **m**,**q**) Z-projection of preformed Syn assemblies (oligomers and fibrils) treated with OleA for two different times (Syn 48 h + OleA 24 h and Syn 48 h + OleA 5d) and (Syn 168 h + OleA 8 h and Syn 168 h + OleA 5d) by immunostaining (red) and GM1 staining (green) on SH-SY5Y cell membrane. (**l**,**p**, and **n**,**r**) FRET analysis between GM1 staining and aggregate immunostaining.

To assess whether Syn amyloid species grown in the presence of OleA modified their propensity to interact with the cell membrane, we performed a sensitized FRET analysis between Alexa-488 GM1 fluorescence and Alexa-546 immunofluorescence on Syn aggregates. We found that both Syn oligomers and fibrils interacted with the cell membrane at the lipid raft level, as shown by the aggregate-GM1 co-localization (Fig. 8a,c) and by FRET efficiency (Fig. 8b,d). Such interaction was drastically reduced when the cells were treated with Syn aggregates grown in the presence of OleA (Fig. 8e,f,g,h). Then, SH-SY5Y cells were treated for 48 h with preformed Syn assemblies exposed for short (Syn 48 h + OleA 24 h, Syn 168 h + OleA 8 h) or long (Syn 48 h + OleA 5d, Syn 168 h + OleA 5d) time to OleA before supplementation to the cell medium. Confocal images and FRET analysis revealed that short treatment of the aggregates with the polyphenol did not alter significantly the ability of the Syn 48 h and Syn 168 h samples to interact with the membrane at the level of the GM1 component of lipid raft domains (Fig. 8i,l,m,n), whereas a prolonged exposure reduced significantly oligomers, but not fibrils, interaction with the cells (Fig. 8o,p,q,r), in agreement with the results of the toxicity experiments reported in the preceding section.

## Discussion

In this study, we show that OleA interferes with Syn fibrillation in a dose-dependent manner, with the optimal 1:10 Syn/OleA ratio; the inhibition is due, apparently, to OleA interaction with Syn monomeric and oligomeric species, an effect similar to that reported for other polyphenols^64,65^. We also report that OleA reduces the toxicity of Syn amyloid aggregates, favouring the growth of harmless off-pathway oligomers. In fact, Syn aggregates grown in the absence or in the presence of OleA, displayed a very different ability to interact with the plasma membrane and to induce sufferance and oxidative stress in exposed cells. Recently, the ability of Syn to self-propagate and spread progressively between interconnected brain regions via a cell-to-cell transmission in PD model and patients has been repeatedly reported and is increasingly accepted as a central mechanism in the development of the disease^66,67^. In view of this notion, several new therapeutic strategies to combat PD are aimed at inhibiting not only Syn misfolding and aggregation, but also the prion-like interneuronal transport of aggregation seeds^22^. In this context, our findings showing a decreased interaction of the OleA/Syn complex with GM1-enriched sites at the cell membrane and the OleA interaction with preformed Syn aggregates (notably oligomers and prefibrillar assemblies), converting them into stable and inert species are of interest. Overall, these data suggest that the polyphenol, besides interfering with the aggregation path, also hinders the association of Syn and/or its aggregates with the cell membrane, presumably, by generating new species with different surface properties. Our findings are of importance in the evaluation of the potential use of OleA in PD treatment, suggesting that OleA could be beneficial not only by interfering with toxic aggregation of Syn and by favouring aggregate disassembly in non-toxic species, but also by hampering their self-propagation.

To better elucidate the mechanism of OleA effects at the molecular level, we investigated the modifications of the structure and cytotoxicity of Syn in different aggregated states arising in the presence of OleA. Syn-OleA interaction was probed by several biophysical and biochemical techniques. We found that OleA does not modify the secondary structure of monomeric Syn and its chromatographic behaviour (retention time in RP and elution volume in GF). On the other hand, the immediate appearance, in the protein/OleA mixture, of a late eluting species in RP (51.2 min), yet earlier than monomeric Syn in GF, clearly indicates that the polyphenol exerts an instantaneous effect on Syn, inducing the formation of a new species (see Figs 4 and 5, for its characterization). The abundance of this species appeared to be dependent on incubation time and OleA concentration, however it did not evolve into fibrils; overall, the structural features of this species allow to classify it as a population of off-pathway oligomers^28,68^. Interestingly, when OleA was added to the aggregation mixture during the elongation phase, the growing aggregates (oligomers and protofibrils) were apparently solubilised into species recalling the off-pathway oligomers formed when OleA was present since the beginning of aggregation. The harmless off-pathway oligomers appeared characterized by a heterogeneous morphology, even though mainly spheroidal. Their secondary structure was substantially random, yet in the presence of a minor content of beta-sheet. Of note, the proteolysis mapping experiments on off-pathway oligomers showed that the NAC and C-terminal regions of Syn are most persistent in solution and suggested a high affinity of OleA for specific regions of Syn. In fact, OleA stabilized the NAC and C-terminal region and hindered the long-range interactions favouring amyloid aggregation.

The limited proteolysis data provided important information on Syn dynamics and structural rearrangement during the aggregation process in the presence or in the absence of OleA. Actually, Syn displayed increased resistance to proteolysis as far as the aggregation process proceeded; this is not surprising, when one considers that, as reported for other amyloid forming proteins, during aggregation the Syn polypeptide chain is progressively embedded into the highly ordered, compact fibrillar structure^58,69^. In the presence of OleA, Syn exhibited a reduced tendency to aggregate and a concomitant increased sensitivity to proteolysis, whereas, the off-pathway oligomeric species eluting at RT 51.2 min were proteolysis-resistant. To better describe this finding, the Syn species were classified as PK-sensitive, PK-resistant and PK-resistant off-pathway oligomers (Fig. S3). The first fraction contained the soluble components of the aggregation mixture; the PK-resistant Syn contained the pre-fibrillar and fibrillar species in equilibrium with the soluble ones; the PK-resistant off-pathway oligomers fraction contained the protease-resistant molecular species eluting at RT 51.2 min. At the beginning of the aggregation (Fig. S3a), the most populated fraction was the PK-sensitive one. As expected, as far as aggregation proceeds, the PK-resistant population increased, at the expense of the PK-sensitive fraction that decreased inversely (Fig. S3b,c). However, when Syn aggregation was carried out in the presence of OleA, the PK-sensitive fraction was stabilized, preventing the formation of the PK-resistant Syn species, while the PK-resistant off-pathway oligomers were increasingly produced over time. The same approach was used to understand (i) whether the same partitioning event between PK-sensitive and PK-resistant species resulted from aggregate disassembly by OleA added at the elongation and plateau phases and (ii) to define Syn conformers preference of OleA. For these reasons, we carried out limited proteolysis on samples enriched either in on-pathway oligomers aged 48 h or on fibrils aged 168 h. ThT measurements clearly showed that OleA decreases fibril formation, when added to a solution enriched in oligomers. In this case, the PK-resistant fraction strongly decreased, while the PK-sensitive Syn increased and the PK-resistant off-pathway oligomers were grown (Fig. S4a). These data confirm that the oligomeric species in the mixture do interact with OleA, giving rise to off-pathway oligomers (PK-resistant RT 51.2) and to a stabilized form of monomeric Syn (PK-sensitive). Moreover, the species populating this sample showed a membrane activity and cytotoxicity that decreased as far as the incubation with the polyphenol was prolonged. In parallel, we observed a concomitant decrease in ROS levels, one of the events associated to amyloid toxicity, which, in this case, was assigned to OleA re-modelling of the growing assemblies, that, eventually, showed different surface properties. In the case of pre-formed fibrils, the ThT data showed a modest effect of OleA. However, the limited proteolysis experiments suggest that the polyphenol induces the formation of the PK-resistant species eluted at RT 51.2 with reduction of PK-resistant Syn, whereas monomeric PK-sensitive Syn was scarcely affected (Fig. S4b). Furthermore, the toxicity of pre-formed fibrils treated with the polyphenol was not significantly affected even after a prolonged treatment (5 days), yet in the presence of a significant reduction of oxidative stress. The reduced cellular levels of ROS could result from the radical scavenging properties of OleA in accordance with previous studies describing the role of the antioxidant properties of this polyphenol in the prevention of amyloid assembly cytotoxicity^70,71^. However, in this case, aggregate toxicity persisted because OleA failed to reduce the membrane activity of the pre-formed fibrils treated with the polyphenol.

In conclusion, our data support the existence of parallel mechanisms of action of OleA. We hypothesized that OleA interacts differently with the previously reported interconverting conformers of monomeric Syn in solution^72^, stabilizing the best-fitting conformation. Moreover, OleA might select the most prone-to-aggregation conformation, confining it into a non-toxic off-pathway oligomer. This view can be supported by the faster conversion of the aggregation-prone monomeric Syn into off-pathway oligomers in the presence of OleA, during the early elongation phase; the latter normally is populated by aggregation-prone monomeric Syn and early on-pathway oligomers, that become solubilised and stabilized in the presence of OleA. At the plateau phase, the effect of OleA on pre-formed fibrils is different, since OleA did not solubilize the fibrils but converted them into smaller assemblies that eluted in RP-HPLC as the off-pathway oligomers grown from aggregation prone Syn. In addition, at molecular level, OleA stabilized the NAC and C-terminal regions of Syn, preventing the long-range and hydrophobic interactions that favour amyloid aggregation^72,73^.

Taken together, our data support the potentiality of polyphenols and similar compounds as neuroprotective agents extending the beneficial properties of olive polyphenols or their molecular scaffolds as promising candidates for long-term nutraceutical treatment of PD. The latter could reduce and delay aging-associated neurodegeneration in sporadic PD limiting the need of the symptomatic pharmacological treatment, the only one presently available, in the expectation that new therapies, such as cell therapy, will improve their efficacy in large number of patients. Moreover, our analysis of the structural and biological features of the Syn oligomeric species grown at different aggregation conditions provides an useful contribution to better understand, at the molecular level, their neurotoxicity in neurodegeneration, as issue of particular relevance in PD pathogenesis.

## Electronic supplementary material

Supplementary Information
