# Supplementary material for: Oleuropein aglycone stabilizes the monomeric α-synuclein and favours the growth of non-toxic aggregates
Source: Sci Rep. 2018 May 29;8:8337. doi: 10.1038/s41598-018-26645-5 (PMC5974307; doi:10.1038/s41598-018-26645-5)
Supplement: Supplementary file 1 — Supplementary Information [file 41598_2018_26645_MOESM1_ESM.pdf]

Supplementary Information

Oleuropein aglycone stabilizes the monomeric α-synuclein and favors the growth of non-toxic aggregates

Luana Palazzi[1§], Elena Bruzzone[2§], Giovanni Bisello[1], Manuela Leri[2,3], Massimo Stefani[2], Monica Bucciantini[2] and Patrizia Polverino de Laureto[1]

[1]*Department of Pharmaceutical Sciences, CRIBI Biotechnology Centre, University of Padova, Italy*

[2]*Department of Biomedical, Experimental and Clinical Sciences, University of Firenze, Italy*

[3]*Department of Neuroscience, Psychology, Drug Research and Child Health, University of Firenze, Italy.*

§ These authors contributed equally to this work

To whom correspondence should be addressed: Patrizia Polverino de Laureto/Monica Bucciantini

Table S1. Molecular masses of fragments purified by RP-HPLC (Fig. 3) after limited proteolysis of Syn by proteinase K (PK), in the absence or in the presence of OleA during aggregation. The term ox refers to oxidation (+ 16 Da).

RT [(a)] *(min)*	*Molecular Mass (Da)* *Found* [(b)]	*Calculated* [(c)]	*Peptide species*
13.3	2909.69±0.53	2909.29	44-72
14.3	1558.09±0.43	1557.84	73-89
	2615.15±0.42	2615.03	31-56
14.8	1800.89±0.46	1800.97	39-56
	3870.88±0.37	3870.42	18-56
	2308.95±0.15	2309.21	1-22
15.4	5201.92±0.57	5185.51	95-140 + 1ox
18.6	4257.34±0.48	4256.87	31-72
19.0	5185.73±0.51	5185.51	95-140
	5228.49±0.36	5227.90	39-92
19.6	5390.06±0.62	5389.74	93-140
	5633.01±0.81	5633.00	90-140
21.5	6546.88±0.01	6547.03	80-140
21.8	5370.26±0.80	5370.11	20-72
23.9	7205.74±1.06	7173.74	73-140 + 2ox
27.6	5679.41±0.41	5662.62	1-56 + 1ox
28.3	7173.94±0.56	7173.74	73-140
28.5	7053.64±0.75	7053.00	18-89
29.1	3200.27±0.12	3200.60	57-89
29.3	5662.07±0.20	5662.62	1-56
29.4	7304.74±0.54	7304.46	1-72
29.5	3443.37±0.25	3443.90	39-72
29.9	7931.42±0.31	7931.17	1-79
30.2	8815.18±0.03	8815.58	57-140
30.3	5797.55±0.07	5797.61	31-89
30.5	8166.49±0.49	8166.28	9-92
31.2	8845.89±0.56	8845.20	1-89
32.1	9089.22±0.97	9088.46	1-92
35.0	14461.12±0.53	14460.19	1-140

[(a)] Peptides are listed in order of retention time (RT).
[(b)] Experimental molecular masses determined by ESI-QTOF-MS.
[(c)] Molecular masses calculated from Syn amino acid sequence.

Table S2. Chemical characterization of fragments corresponding to the peaks of the chromatograms relative to the proteolysis of Syn/OleA oligomers (Fig. 5a) and Syn as monomers (Fig. 5b) with trypsin. The term ox indicates the presence of one oxidation (+16 Da). Two species are indicated, when they have the same molecular weight.

RT51.2 + trypsin			
RT [(a)] *(min)*	*Molecular Mass (Da)* *Found* [(b)]	*Calculated* [(c)]	*Peptide species*
13.2	1294.70±0.03<br>1523.82±0.01	1294.69<br>1523.83	46-58<br>44-58/46-60
13.9	829.43±0.01<br>950.53±0.01<br>4287.89±0.12	829.43<br>950.51<br>4287.88	24-32<br>35-43<br>59-102
23.3	6418.99±0.02	6418.86	81-140
29.9	5564.98±0.11	5565.33	46-102
30.2	8558.13±0.02<br>8574.30±0.02	8558.29<br>8558.29	59-140<br>59-140 + 1ox

Syn + trypsin			
RT [(a)] *(min)*	*Molecular Mass (Da)* *Found* [(b)]	*Calculated* [(c)]	*Peptide species*
11.2	1058.55±0.01	1058.57	24-34/22-32
11.5	1287.72±0.09	1287.71	22-34
12.4	872.47±0.01<br>1071.60±0.01	872.46<br>1071.59	13-21<br>11-21
13.2	1523.84±0.01	1523.83	44-58/46-60
13.3	1294.70±0.01<br>1752.93±0.01	1294.69<br>1752.97	46-58<br>44-60
13.8	1179.66±0.01<br>1991.20±0.01<br>2112.50±0.02<br>2220.03±0.03	1179.65<br>1991.07<br>2112.15<br>2220.21	35-45/33-43<br>24-43<br>11-32<br>22-43/24-45
14.1	950.51±0.01	950.51	35-43
14.9	2156.20±0.01	2156.18	59-80
15.3	769.56±0.01<br>1477.76±0.01	769.35<br>1477.78	1-6<br>81-96
17.7	6450.98±0.05	6418.86	81-140 + 2ox
19.1	4958.15±0.01	4958.20	97-140
19.6	4288.76±0.01<br>4303.77±0.05<br>4830.06±0.01<br>4846.04±0.01	4288.43<br>4288.43<br>4830.03<br>4830.03	103-140<br>103-140 + 1ox<br>98-140<br>98-140 + 1ox
23.2	6418.94±0.01<br>6434.93±0.01	6418.86<br>6418.86	81-140<br>81-140 + 1ox
28.2	3434.90±0.01<br>3664.05±0.01	3434.90<br>3664.18	46-80<br>44-80
30.2	8558.11±0.01<br>8574.30±0.01	8558.29<br>8558.29	59-140<br>59-140 + 1ox
32.0	10064.93±0.01<br>10080.93±0.01	10065.02<br>10065.02	44-140<br>44-140 + 1ox

[(a)] Peptides are listed in order of retention time (RT).
[(b)] Experimental molecular masses determined by ESI-QTOF-MS.
[(c)] Molecular masses calculated from Syn amino acid sequence.

Table S3. Molecular masses of the main fragments purified by RP-HPLC (Fig. 5c-f) after proteolysis of RT 51.2 by proteinase K (PK) for 5 (Fig. 5c,d) and 70 min (Fig. 5e,f) with and without Gnd-HCl. The term ox indicates the presence of oxidation (+16 Da).

RT[(a)] *(min)*	*Molecular Mass (Da)*		*Peptide species*
	Found[(b)]	*Calculated*[(c)]	
11.8	1272.88±0.01	1272.67	18-30
12.0	644.44±0.05<br>607.36±0.09	644.35<br>607.25	73-79<br>136-140
13.3	1659.16±0.05	1658.88	57-72
13.7	1772.99±0.03	1772.67	126-140
14.1	1557.84±0.01	1557.84	73-89
14.3	2615.57±0.01	2615.03	31-56
14.6	3799.23±0.01<br>1800.96±0.01	3799.34<br>1800.97	19-56<br>39-56
14.8	5665.20±0.23<br>5421.82±0.28	5633.00<br>5389.74	90-140 + 2ox<br>93-140 + 2ox
15.2	5202.50±0.83	5185.51	95-140 + 1ox
15.6	5649.75±0.11<br>5405.83±0.47	5633.00<br>5389.74	90-140 + 1ox<br>93-140 + 1ox
17.1	1880.21±0.02	1879.99	1-18
17.8	5649.53±0.01<br>5405.40±0.01	5633.00<br>5389.74	90-140 + 1ox<br>93-140 + 1ox
19.1	5185.85±0.79	5185.51	95-140
19.8	5632.83±0.07<br>5389.27±0.20	5633.00<br>5389.74	90-140<br>93-140
27.7	5170.92±0.08<br>3065.45±0.01	5170.02<br>3065.61	5-56<br>1-30
28.2	7173.81±0.05	7173.74	73-140
28.8	5662.84±0.20<br>3200.56±0.36	5662.62<br>3200.60	1-56<br>57-89
29.0	7304.84±0.16	7304.46	1-72
30.0	8815.56±0.21	8815.58	57-140
51.2	14461.20±0.69	14460.19	1-140

[(a)] Peptides are listed in order of retention time (RT).
[(b)] Experimental molecular masses determined by ESI-QTOF-MS.
[(c)] Molecular masses calculated from Syn amino acid sequence.

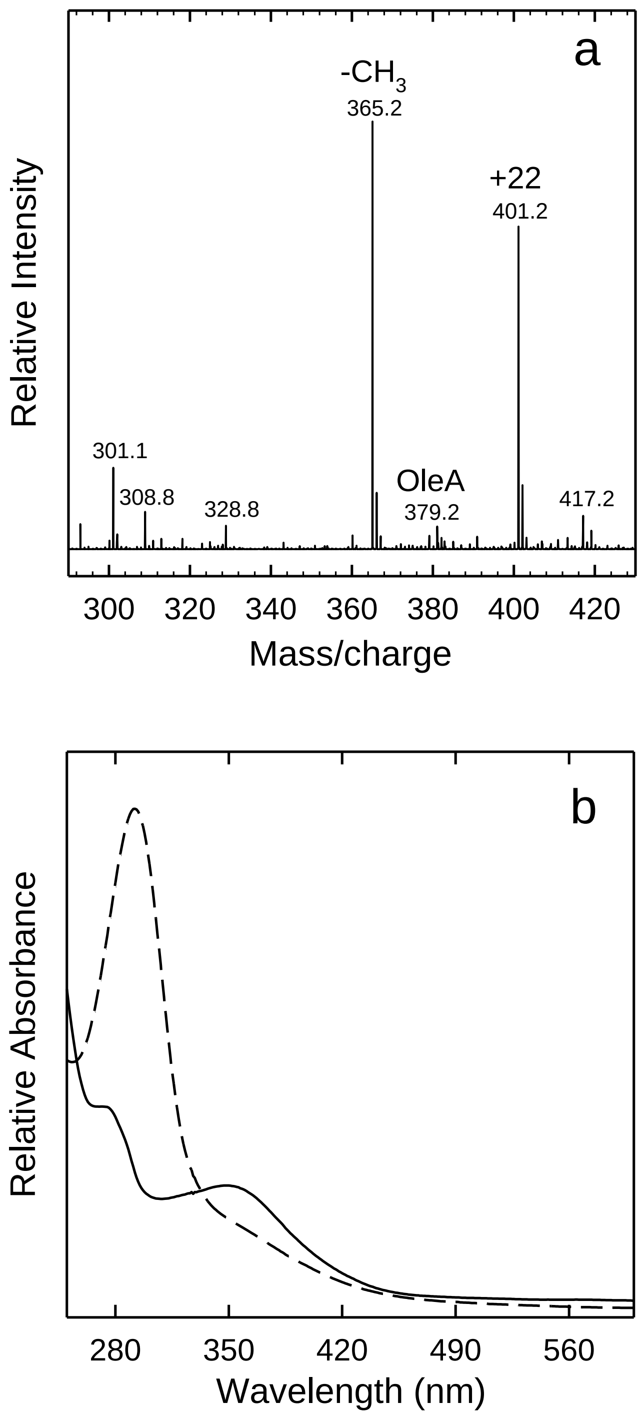

Figure S1. Chemical characterization of OleA. OleA samples were analysed by ESI-MS in positive mode (Fig S1a). The main products are the demethylated form at m/z 365.2 Da (-CH_3, -14 Da) and the adduct with sodium at 41.2 Da (+ 22 Da). In Fig S1b, the UV-Vis spectra of OleA samples incubated in PBS buffer for 0h (black continuous line) and 168h (dashed line) are reported.

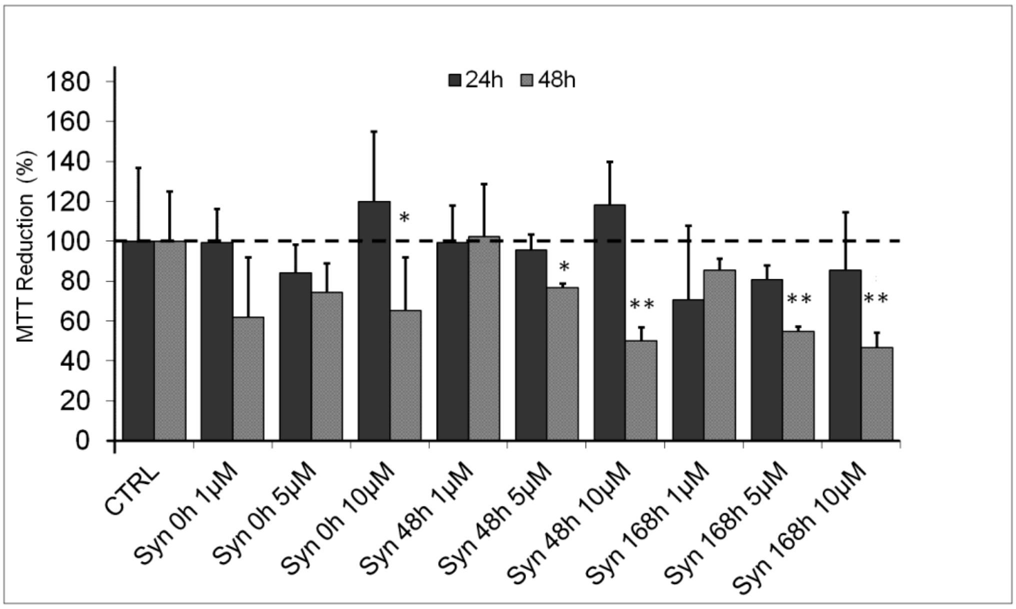

Figure S2. Cytotoxicity of Syn aggregates at different fibrillation times. SH-SY5Y cells were treated for 24 h or 48 h with Syn as monomer, oligomer or fibril, obtained after 0 h, 24-48 h or 168 h of aggregation, respectively. Oligomers and fibrils showed dose-dependent cytotoxicity after 48 h of cell incubation. Error bars indicate the standard deviation of independent experiments carried out in triplicate. Student's *t*-test: *$p < 0.05$; **$p < 0.01$ versus control.

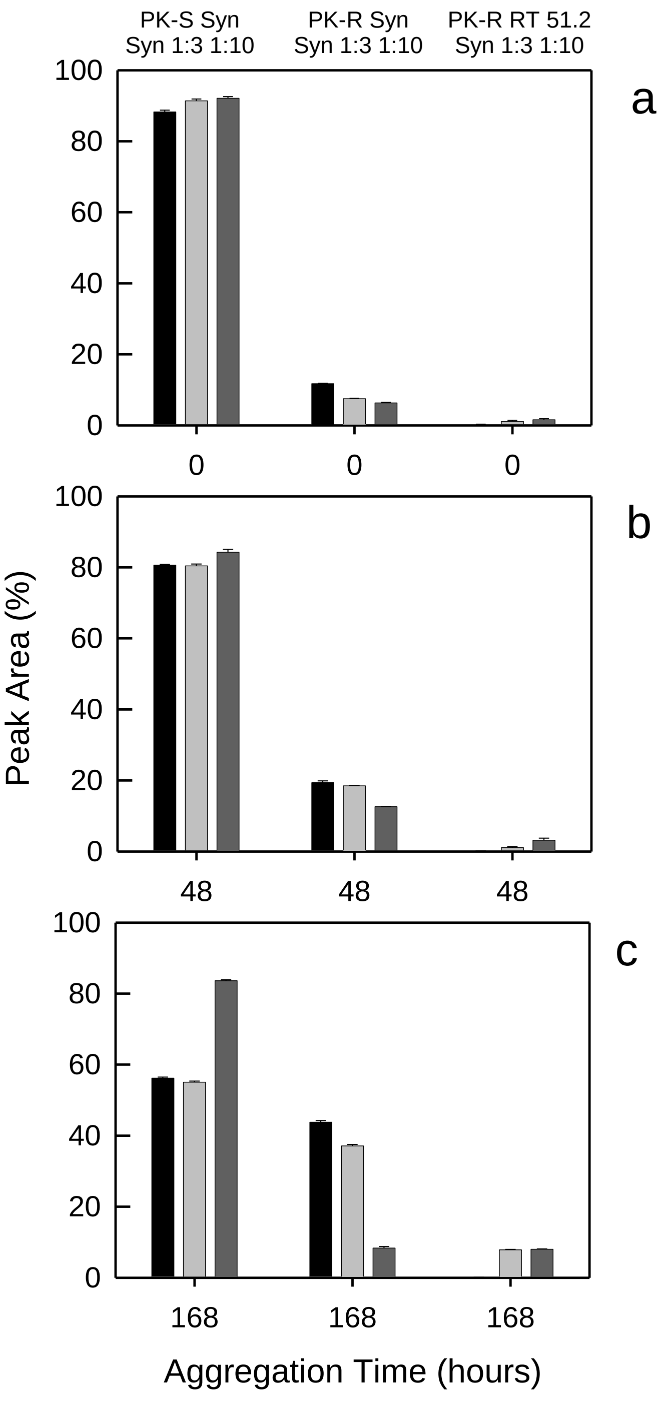

Figure S3. Quantification of the different population of Syn coexisting in the aggregation mixture in the absence or in the presence of OleA, calculated by the limited proteolysis experiments shown in Fig. 3. The extent of each population was evaluated from the area of the chromatographic peaks relative to the purification of the proteolysis mixtures of Syn with proteinase K (PK) during aggregation. Peaks areas relative to proteolysis (a) at 0 h; (b) at 48 h; (c) at 168 h of Syn incubation. PK-S Syn consists of the fragments produced by PK-proteolysis; PK-R Syn represents undigested Syn and PK-R RT 51.2 represents undigested off-pathway oligomers.

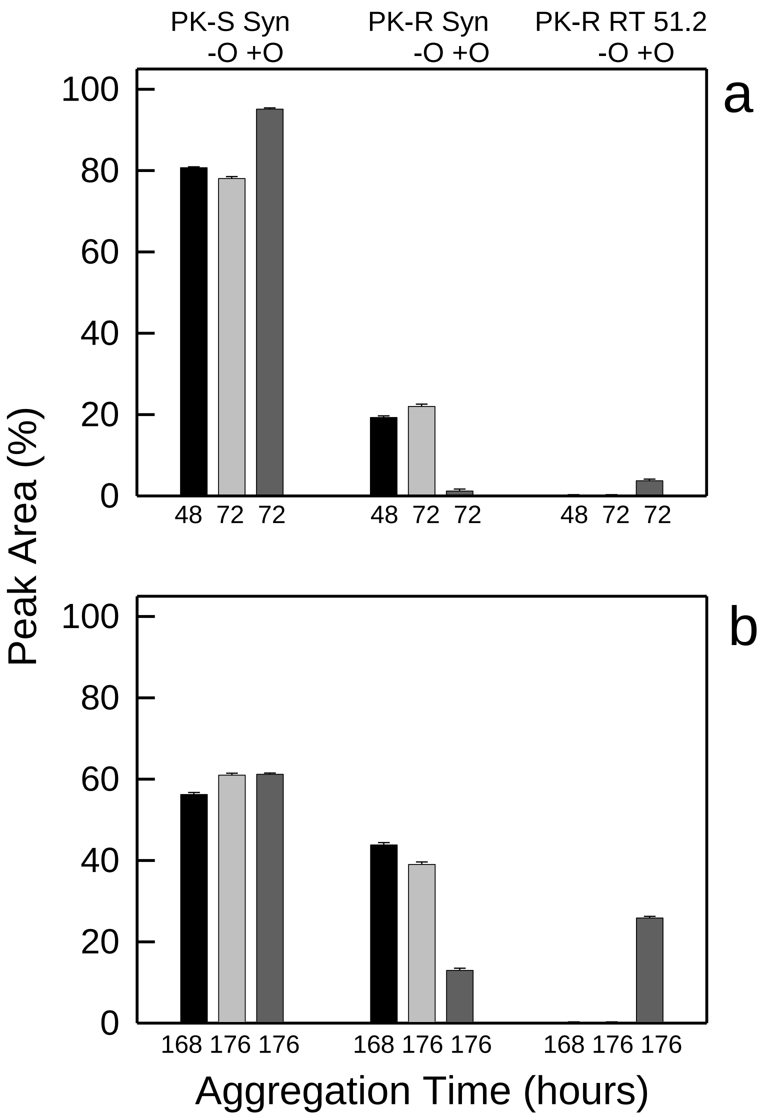

Figure S4. Quantification of the different population of aggregated Syn arising during disaggregation following addition of OleA, calculated by the limited proteolysis experiments shown in Fig. 7c-h. The amount of each population was evaluated from the area of the chromatographic peaks relative to the purification of the mixtures of Syn arising from PK proteolysis during disaggregation. (a) Peak areas obtained from the purification of the proteolytic oligomeric-enriched mixture (48h), and the same mixture after further 24h-incubation in the presence (+O) or in the absence (-O) of OleA. (b) Proteolysis of the fibril-enriched mixture (168h) and the same mixture after further 8h-incubation in the presence (+O) or in the absence (-O) of OleA. PK-S Syn represents Syn fragments; PK-R Syn represents undigested Syn and PK-R RT 51.2 represents undigested late-eluting aggregates.
